# Preeclampsia: Molecular Mechanisms, Predisposition, and Treatment

**DOI:** 10.1155/2012/145487

**Published:** 2012-05-29

**Authors:** Nicolaos Vitoratos, Nicolaos Vrachnis, Christos Iavazzo, Maria Kyrgiou

**Affiliations:** ^1^2nd Department of Obstetrics and Gynecology Aretaieio Hospital, University of Athens, 11528 Athens, Greece; ^2^Department of Obstetrics and Gynaecology, Hammersmith Hospital, Queen Charlotte's and Chelsea Hospital, London W12 0HS, UK

Preeclampsia is a disorder characterised by vascular endothelial dysfunction and vasospasm that occurs after 20 weeks of gestation till 4–6 weeks postpartum. The global incidence of preeclampsia has been estimated at 2–14% of all pregnancies. Despite the advances made in the field, research is still organised to clarify the possible molecular mechanisms or predisposing factors of preeclampsia as well as the treatment options for such a significant disorder. Papers were selected on the basis of fundamental research ideas or reviews in the field. This special issue is comprising of seven papers which are focused on the better understanding of preeclapsia.

One of the papers deals with the correlation of preeclampsia with hypoxia, thrombosis, and inflammation. The authors suggest that there is no accurate test for predicting preeclampsia. The role of markers such as sFLT, sEng, products of fetal and placental origin, markers of renal or endothelial damage, or markers of oxidative stress is presented by acting as secondary pathways to the pathophysiological changes that precede the clinical onset of preeclampsia. A combination of such markers is proposed in order to increase the detection accuracy earlier in the pregnancy and hopefully allow for more effective prophylactic strategies.

Another paper sought to validate the use of urinary podocyte (podocyturia) as a single diagnostic marker in preeclampsia and in differentiating from other high-risk pregnancy states with similar presentations. The researchers discovered that podocyte loss is present not only in preeclampsia but in other high-risk pregnancy states. In addition, podocyturia was not found in a majority of patients diagnosed with preeclampsia. So, they realized that their findings had relatively low sensitivity and specificity, but they proposed further research regarding the predictive value of podocyturia in preeclampsia in larger studies.

The authors of one of the paper investigated whether there is an association between angiotensin converting enzyme (ACE) insertion/deletion (I/D) polymorphism and preeclampsia in 236 pregnant women. They showed that there was significant difference in terms of genotype distribution between preeclampsia and controls, while it was not found any difference in allele frequency for ACE I/D polymorphism. A possible reason for the inconsistency could be the genetic basis that caused different susceptibilities among different populations.

Moreover, one paper is mentioning that both women and children exposed to preeclampsia exhibit an adverse vascular phenotype, a propensity to subclinical atherosclerosis, and increased risk of adverse cardiac and vascular events in future life. They suggest that further studies into the mechanisms such as vascular dysfunction underlying the altered cardiovascular phenotype might provide unique insight into pathophysiological or molecular links between preeclampsia and cardiovascular disease which may direct us to novel treatment strategies for both conditions. Improvement in vascular function is also proposed as a valuable intermediate end point in studies aiming to reduce risk in this potentially young and generally asymptomatic population before the onset of clinical disease.

F. J. Valenzuela and colleagues have recently reviewed some polymorphisms in important candidate genes involved in different pathogenic mechanisms related to preeclampsia and concluded that various studies in different populations have identified maternal polymorphisms associated with preeclampsia through candidate gene approaches.

Luizon and colleagues with a Letter to the Editor add to the paper of F. J. Valenzuela et al. by further referring to candidate genes related to angiogenesis and endothelial dysfunction in preclampsia performed in the Brazilian population. Specifically, genotypes and haplotypes formed by polymorphisms of VEGF, eNOS, and MMP-9, along with an example of the interaction among these genes in the prediction of preeclampsia provide additional information with clinical relevance to its susceptibility.

An additional paper is a review of the molecular mechanisms which are contributing to the pathogenesis of preeclampsia. Altered angiogenic balance, systemic inflammation, dysregulation of Renin-Angiotensin system, and placental hypoxia or ischemia are mechanisms leading to the pathogenesis of preeclampsia. However, it is unknown whether the mechanisms act independently or have synergistic effects.

